# The Effect of Natural and Synthesised Zeolites on Cement-Based Materials Hydration and Hardened State Properties

**DOI:** 10.3390/ma16165608

**Published:** 2023-08-13

**Authors:** Giedrius Girskas, Ina Pundienė, Jolanta Pranckevičienė

**Affiliations:** Institute of Building Materials, Vilnius Gediminas Technical University, Linkmenų Str. 28, LT-10223 Vilnius, Lithuania; giedrius.girskas@vilniustech.lt (G.G.); jolanta.pranckeviciene@vilniustech.lt (J.P.)

**Keywords:** synthetic zeolite, electric conductivity, pH, rheology, compressive strength, absorption

## Abstract

The synthesis of zeolites from difficult-to-utilise waste materials facilitates the creation of more financially attractive and efficient synthetic zeolites. These can be incorporated into construction materials, resulting in a reduction in cement usage and the production of superior, clean, and sustainable construction materials. The potential to enhance the hydration rate of fresh cement paste by substituting up to 10% of the cement with two synthetic zeolites—one commercially produced and the other synthesised from waste and natural zeolite—was explored. Due to a higher Al/Na ratio, newly sintered waste-based zeolite possesses six times higher electrical conductivity compared to industrially produced 4A zeolite and more than 20 times higher electrical conductivity compared to natural zeolite. As the sequence of this fact, substituting up to 10% of the cement with AX zeolite cement paste accelerates the maximum heat release rate time and increases the total heat by 8.5% after 48 h of hydration. The structure, compressive strength, and water absorption of the hardened cement paste depends on the Al/Na ratio, pH, and electrical conductivity values of the zeolite used. The findings revealed that AX zeolite, due to presence of mineral gibbsite, which speeds up hydration products, such as CSH development, increases the compressive strength up to 28.6% after 28 days of curing and reduces the water absorption by up to 1.5%. Newly synthesised waste-based AX zeolite is cheap because its production is based on waste materials and is mostly promising due to superior properties of created construction materials compared to the other presented zeolites.

## 1. Introduction

Zeolites are widely used around the world by different industries due to their unique properties, such as ion exchange, pozzolanic activity, absorption properties, lightness, safety. Not all natural zeolites can be synthesised, and not all synthetic zeolites have natural zeolite equivalents. Both natural zeolites, of which there are 50 known types (clinoptilolite, modernite, phillipsite, ernonite, chabazite, etc.), and specially developed synthetic zeolites (A, X, Y, L, ZSM-5, etc.) are widely used [1]. At least 214 species of synthetic zeolites are known today. Synthetic zeolites are mainly used in catalysts, gas separation and ion exchange. The zeolite application, agricultural, environmental protection, medicine, and building area industries have been expanding [2]. Zeolites are widely used in finishing products to obtain more even surfaces and faster-setting mortars. Among the benefits are cement paste stability, increased volume, lower fresh concrete segregation, reduce hardened concrete porosity, and increased durability and compressive strength. Zeolite admixtures modify and improve the Portland cement hydration process, effectively changing the physical and mechanical properties of hardened cement pastes [3]. For example, a waste catalyst added to concrete mix at 15% by weight of cement increases the early compressive strength and reduces the cement hydration time [4,5]. Zeolites have a high content of active SiO_2_ and Al_2_O_3_, similar to other pozzolanic substances, and can improve the strength of concrete through the reaction of Ca(OH)_2_ with pozzolans. The partial replacement of cement by selected amounts of zeolite can provide a greater strength increase in concrete compared to concrete without zeolite [6].

Zeolites reduce the content of Ca(OH)_2_ produced in the hydration of cement minerals and thus form hydration products similar to cement hydration products. The pozzolanic reactions of zeolite are rather slow at the beginning of cement hydration reaction; however, they accelerate significantly after 28 days of hydration [7]. The results of studies on early hydration and pozzolanic reaction in samples made of cement and zeolite revealed that the initial hydration of C_3_S accelerates with the addition of zeolites. The kinetic analysis of C_3_S consumption showed that the precipitated layer of C-S-H is thinner and not so dense. The addition of zeolites changes the structural release of C-S-H products [8]. Researchers found that the Ca(OH)_2_ content in the cement mass reduced as a result of pozzolanic reactions in the presence of zeolite, that the hydrated mass surface were increased, and that the diameter of internal pores was reduced [9]. However, the pozzolanic reaction depends on the zeolite structure [10]. The researchers proved that a zeolite admixture reduces the Ca(OH)_2_ content in hardened cement paste. A zeolite admixture also induced the formation of monosulphoaluminates and carboaluminates in cementitious systems [11].

Synthetic zeolites, due to their predictable behaviour, are some of the most promising components of concrete mixes when designing new products (building materials), such as high performance concretes, special concretes able to absorb heavy metals or suppress radiation, and non-autoclave silicate products [12]. Synthetic zeolites allow for a decrease in the weight of structural elements in buildings without compromising strength indicators; however, their use is limited by a rather high price. Synthetic zeolites can be also used as adsorbents or traditional water softeners and detergents [13,14].

Synthetic zeolites can be synthesised from crystal and amorphous materials, from aluminium silicate gels or alkaline metals, or by heating aluminium silicate mineral suspensions, hydroxides, and water. The initial formation of zeolite structures can start only in the presence of water [15,16].

With the growing amount and variety of industrial waste products, there is a wide choice of raw materials and by-products, such as glass scraps, various slags and clays, coal burning ash, fly ash, by-product of the extraction of non-aggregate minerals, and chemical industry waste [17,18,19,20,21], for zeolite synthesis.

By reusing production waste or using natural mineral materials instead of reagent raw materials, it is possible to reduce the price of zeolite synthesis [22,23]. The synthesis of Zeolite A was achieved by researchers [24] by burning Egyptian kaolin at 800 °C. Zeolites NaPl, analcime, and chabazite [25] can be synthesised through the reaction of fly ash and NaOH in solution or from coal ash [26], with the final products being ZFA0, ZFA32, ZFA42, ZFA76, and ZFA88. Phosphogypsum production residues, such as aggressive technogenic substance, contaminated with fluorides, or AlF_3_ manufacture waste can be used for zeolite synthesis, based on the reaction of sodium hydroxide and amorphous silica gel in aqueous medium. The obtained products are analcyme and hydrosodalite [27,28]. It was concluded that additional amount of aluminium had a positive effect on the amount of analcyme and hydrosodalite in the synthesised products. Industrial waste based on aluminosilicate compounds are suitable initial materials for the preparation of zeolites. The main properties and application fields of such types zeolites are similar to those of commercial zeolites made from reagent materials [22,23,29]. Synthesised waste-based zeolites are used for the improvement of compressive strength, water permeability, and corrosion resistance; however, the challenges related to the slowing down of cement hydration and low initial strength by using zeolites remain unsolved [22].

The aim of this paper is to identify the applicability of synthesised difficult-to-utilise technogenic waste (aluminium fluoride (AlF_3_) manufacture waste product)-based zeolite and compare it to that of industrially produced zeolite 4A and natural zeolite as possible supplementary cementitious material in cementitious structures. Furthermore, the objectives of this study are to evaluate the pH, electrical conductivity, rheological properties, the hydration parameters of cement pastes, and to test the early strength properties of hardened modified cement pastes after different cement hydration times and assess the benefits of the newly synthesised waste-based zeolite. The objectives of this study are to evaluate the pH, electrical conductivity, rheological properties, and hydration parameters of cement pastes, which affect also the tested early strength properties of hardened modified cement pastes after different cement hydration times.

## 2. Materials and Methods

### 2.1. Materials

Portland cement CEM I 42.5 R was used for the tests. The physical and mechanical properties as well as the chemical and mineral composition of the binding materials are presented in Table 1. Cementitious systems were modified by synthesised zeolite, produced from the following materials: aluminium fluoride (AlF_3_) manufacture waste product; sodium hydroxide; aluminium hydroxide.

All these material were carefully mixed until a homogeneous mass is obtained. Afterwards, 180 g of water is added, and the mixing continues until a homogeneous suspension is obtained. The mixing is followed by a synthesis, which lasts for 1–3 h at 95–105 °C. The synthesis products are filtered through a Buchner funnel, and the excessive sodium hydroxide is washed out. The obtained products are dried at 60–100 °C for 24 h. Dried products are sieved through a 0.315 mm sieve. Dried and sieved synthesis products are modified with a calcium chloride (CaCl_2_) solution by mixing it at 80 °C for 5 min. The obtained synthetic zeolite is grounded to the size of cement particles.

The chemical compositions of synthetic AX zeolite, 4A, and natural zeolite (clinoptilolite) are given in Table 2. Properties of natural zeolite are as follows: porosity, 34%; density, 2.370 kg/dcm^3^; radionuclide activity, up to 144.5 Bg/kg; conditional surface, ~144 m^2^/g; mechanical compressive strength, 150 kg/cm^2^; clinoptilolite content, up to 90%; abrasion, up to 4%; ion shift susceptibility, 1.5 mg.ekv/g; and humidity, 4%. A polycarboxylate resin-based plasticiser was also used in the sample preparation. The plasticiser characteristics are as follows: resin concentration in the solution, 36.1%, pH value, 5.05; electrical conductivity, 1.480 mS/cm; and solution density, 1040 kg/m^3^.

### 2.2. Composition of the Mixes

Fresh cement paste and hardened cement paste samples were formed taking into consideration the effect of cement replacement by zeolite on the properties of cementitious systems.

The cement paste compositions are given in Table 3. The first cement paste, CP-0, is the control composition, containing no zeolite admixtures. The synthetic zeolite obtained from aluminium fluoride manufacture waste product was used to replace the cement in mix compositions CPAX-5 and CPAX-10 at 5% and 10% by weight of cement, respectively. Compositions CPN-5 and CPN-5 contained natural zeolite. Compositions CP4A-5 and CP4A-10 contained zeolite 4A. The ratios of water and solid substance (W/C) of all mixes are given in Table 3. W/C ratio differs slightly from 0.27 to 0.28.

Cement and zeolite admixtures were dosed by weight and water, while chemical agents were dosed by volume. Chemical admixtures were mixed with water and afterwards mixed with cement and zeolite admixtures. Cement pastes were prepared in a forced action mixer, the mixing time was 90 s.

Concrete beams (40 × 40 × 160 mm) were made after cement paste consistencies were determined in order to determine the physical and mechanical properties of the hardened cement pastes. The samples were compacted on laboratory vibrating plate for 20 ± 2 s. The samples were kept in forms for 24 h at ambient temperature (20 ± 2 °C), then removed from the moulds and hardened in water at 20 ± 2 °C for 7, 14, or 28 days.

### 2.3. Research Methods

In order to better describe how the type of zeolite used will affect the pH and electrical conductivity values of the cement pastes, the pH and electrical conductivity were determined using a Mettler-Toledo MPC 227, Greifensee, Switzerland, Mettler Toledo AG pH electrode (InLab 410), and the accuracy of the measurement was 0.01. The electrical conductivity electrode (InLab 730) had a measurement range of 0–1000 μS/cm. The pH and electrical conductivity were determined after 1, 2, 3, 4, 6, 8, 10, and 24 h. The proportion of paste was 1:5 (dry matter/water). The measurements of the modified pastes were recorded at 20 ± 0.5 °C temperature. Both the pH and electrical conductivity measurements were performed 3 times for each paste composition. The arithmetic averages of these measurements are presented in the study.

The impact of type f zeolite and its amount on the rheology of cement pastes was measured by vibrational viscometer (SV–10). The modified cement pastes were mixed at 20 ± 0.5 °C. 40–45 mL of cement paste were used for the tests. Plates moving at constant speed were immersed into the container with cement paste to measure its viscosity by resistance force. The rheology test was repeated 3 times for every composition of the tested cement paste, and the arithmetic averages of the three individual measurements are presented in the study.

For the calorimetric measurements, a differential isothermal calorimeter “ToniCAL III” was used. Three mixes with different zeolites were used, but the same cement to zeolite replacement ratio (10% mass of cement) was tested. A control sample with no zeolite was prepared. For the sample preparation, 45 g of distilled water and 100 g of solid substance, in which 10% of the cement was replaced by zeolite (natural zeolite, AX zeolite, and 4A zeolite), was used. All solid components, before testing, were mixed in dry conditions. The test was conduct at 20 °C, and the heat evolution curves were registered during the hydration reaction for up to 48 h.

The hydration kinetic characteristics of the fresh cement pastes were measured using an exothermic profile according to the Alcoa methodology [30]. The heat generated in the exothermic reactions of cement minerals in fresh paste was measured at 20 °C using 1.5 kg samples placed in an insulated 10 × 10 × 10 cm textolite mould. A thermocouple (type T) was embedded in the sample and linked to a data capture system to record the temperature as a function of time.

40 × 40 × 160 mm prisms of hardened cement paste were formed, and the following basic physical and mechanical characteristics were determined: density after 7, 14, 28 days; compressive strength after 7 and 28 days of curing; and water absorption samples hardened for 7, 14, and 28 days.

9 prisms (40 × 40 × 160 mm) of every composition of hardened cement paste were taken to measure water absorption. The water absorption test was conducted after a curing period of 7, 14, and 28 days. After the selected curing period, the samples were dried to the constant mass, weighted, and placed in water for 24 h.

The compressive strength of samples was determined in accordance with EN 12390-3 requirements. The compressive strength tests were performed using an “ALPHA 3-3000 S” compression testing machine. 3 samples of each composition were tested after 7 and 28 days of curing, and the arithmetic averages of the 3 individual measurements are presented in the work.

The structure of the zeolite admixtures and modified hardened cement stone samples (natural zeolite, zeolite 4A, and synthetic zeolite from AlF3 manufacture waste product) was analysed using an scanning electron microscope (SEM JOEL JSM-7600F, JOEL, Tokyo, Japan) with a resolution of 1.5 nm and a magnification of 25 to 1,000,000 times. A total voltage of 10.0 kV was used for the tests, and the surface of the tested samples was covered with gold. X-ray tests were performed using a diffractometer DRON-7 (Zavlab AG, Charkiv, Ukraine) with a Cu anticathode and Ni filter, and an anode voltage of 30 kV, an anode current of 12 mA, and a detector step of 0.02º. The profiles of the diffraction peaks were analysed via peak deciphering achieved by means of a crystallographic database (ICDD). The particle size of the binding material and zeolite additives as well as grain distribution was determined using a laser particle size analyser (CILAS 1090, LabWrench, France, Paris) using a dry and liquid method. Water was used as the dispersion medium, and the content of dry matter in the suspension was 8–12%. Particle size was measured within a 0.01–500 μm interval.

## 3. Results

### 3.1. Zeolite XRD

**Figure 1:**
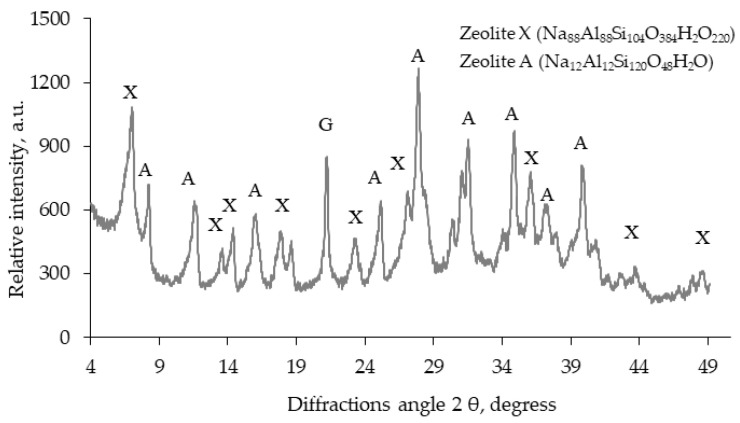
XRD of zeolite modified with CaCl_2_. X: X zeolite modification; A: A modification of the zeolite; G: gibbsite.

X-ray diffraction analysis is widely used for the analysis of the crystal structure in zeolites and other materials, i.e., to identify chemical compounds and their variants, to conduct a quantitative analysis of the compounds, to determine the crystal grating structure in monocrystalline and polycrystalline materials, and to identify grating defects. Figure 1 shows that the synthetic zeolite admixture is made of zeolite X (Na_88_Al_88_Si_104_O_384_H_2_O_220_) (0.757, 0.643, 0.578, 0.443, 0.381, 0.289, 0.240, 0.207, and 0.146 nm) and zeolite A (Na_12_Al_12_Si_120_O_48_H_2_O) (1.250, 0.887, 0.716, 0.411, 0.371, 0.329, 0.292, 0.262, and 0.250 nm) with traces of gibsite (α-Al(OH)_3_).

**Figure 2:**
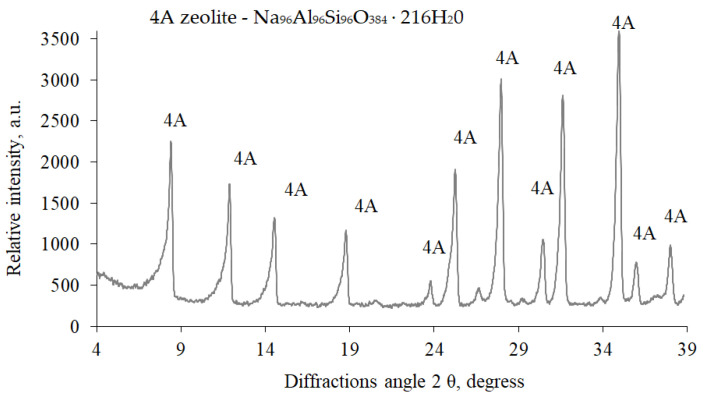
XRD spectrum of modified zeolite 4A.

**Figure 3:**
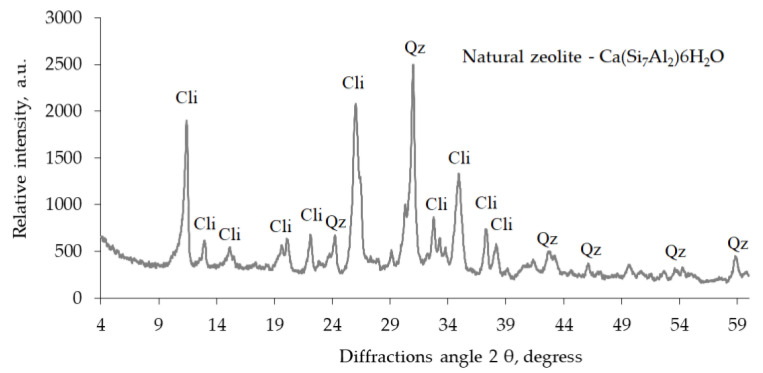
XRD spectrum of natural zeolite. Cli, clinoptilolite; Qz, quartz.

A natural zeolite admixture (clinoptilolite-based) and zeolite ZP-4A admixture (modification 4A) were also used to test the effect of zeolite admixtures on the rheological properties of cement pastes and the physical mechanical characteristics of hardened cement paste. X-ray diffraction analysis showed (Figure 2) that the zeolite ZP-4A admixture is zeolite modification 4A (1.255, 0.866, 0.708, 0.548, 0.434, 0.408, 0.370, 0.341, 0.28, 0.298, 0.289, and 0.75 nm) with the chemical formula Na_96_Al_96_Si_96_O_384_ · 216H_2_O. Natural zeolite (Ca(Si_7_Al_2_)6H_2_O) containing clinoptilolite as the main component was also used as an admixture in a hardened cement paste. X-ray analysis of this zeolite (Figure 3) revealed the prevalence of clinoptilolite (0.897, 0.796, 0.678, 0.526, 0.512, 0.466, 0.397, 0.342, 0.317, 0.312, 0.298, 0.279, 0.274, and 0.253 nm) and quartz (0.426, 0.335, 0.245, 0.243, 0.228, 0.213, and 0.182 nm). The chemical composition of natural zeolite is as follows: SiO_2_, 71.5%; Al_2_O_3_, 13.1%; CaO, 2.1%; MgO, 1.07%; Fe_2_O_3_, 0.9%; TiO_2_, 0.2%; and H_2_O, 11.13%.

Laser particle size analyser was used to determine the size of the cement and zeolite particle size. It should be noted that the biggest particle size, 14.24 μm, was found in the cement, whereas the average particle sizes of natural zeolite and AX zeolite obtained from aluminium fluoride manufacture waste product were very similar, at 11.82 μm and 11.61 μm, respectively. The smallest particle size was found in zeolite 4A, with an average size of 5.38 μm.

### 3.2. Cement Paste pH and Electrical Conductivity

Tests on cement pastes containing different zeolites were conducted in order to see how they affect the pH values of the cement suspensions. A natural zeolite admixture (clinoptilolite-based), synthetic modified zeolite 4A, and synthetic zeolite admixture obtained from aluminium fluoride manufacture waste product were used for the tests. The aim of these tests was to find out how the pH of zeolite and the cement suspensions changes with time. The initial pH values of the plasticising admixture (M-FK), cement, and zeolite suspensions showed that the pH of the plasticising admixture was 5.05 and that the pH of the cement suspension was 12.18. The pH values of the zeolite suspension are as follows: 9.93 for the zeolite admixture obtained from aluminium fluoride manufacture waste product (AX), 7.01 for the natural zeolite (N), and 9.7 for the synthetic zeolite 4A. Despite the highest alkali amount being obtained from the 4A zeolite, the highest pH value belongs to AX zeolite. This can be explained by the presence of X zeolite with high alkali amount and gibbsite quantity.

**Figure 4:**
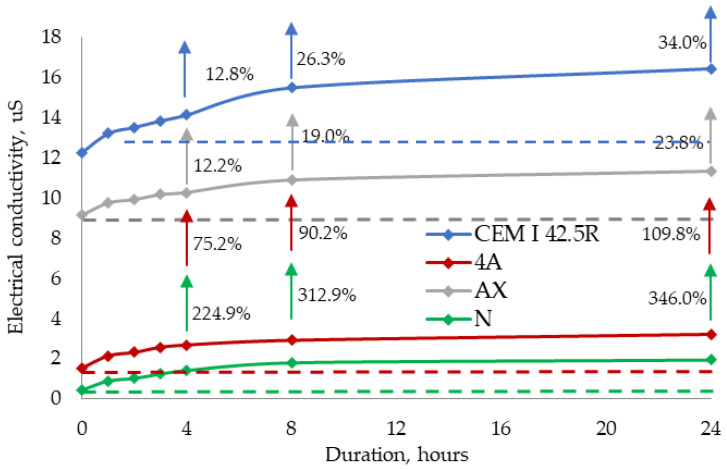
Electrical conductivity kinetics of cement pastes with various zeolites.

The pH kinetics test was conducted for 24 h and showed a slight change in pH values, i.e., after 24 h the pH of the cement suspension changed by 1.4%, and the pH of the zeolite suspension changed from 1.1 to 1.6%. The electrical conductivity kinetics test (Figure 4) showed an increase in the electrical conductivity values during the 24 h. During the first hour of the test, the electrical conductivity values of the cement suspension changed from 12.26 to 13.2 mS, and later, a change of about 0.3 mS per hour was observed. After 8 h, the electrical conductivity was 15.48 mS, and after 24 h, a 34% increase from the initial values was recorded. Sush results are confirmed by research [29].

The initial electrical conductivity of the zeolites was different: 1.53 mS for zeolite 4A, 0.433 mS for natural zeolite NC, and 9.15 mS for zeolite AX. As was pointed out in [31], the solubility of gibbsite in alkaline systems can be increased. This is why the electrical conductivity of zeolite AX was the highest.

After one hour, the electrical conductivity value in synthetised zeolite AX and in 4A zeolite increased by 6.5% and 28%, respectively. The electrical conductivity value in natural zeolite increased from 0.433 to 0.885, i.e., more than twice. Later on, the electrical conductivity values increased by about 0.2 mS per hour, and after 4 h, the electrical conductivity value of the synthetic zeolite 4A increased by more than 75% of the initial value, by 12% for the zeolite AX, and by more than 3.2 times for the natural zeolite NC. After 24 h, the electrical conductivity of the zeolite AX increased from 9.15 to 11.33 mS, from 1.53 to 3.21 mS for the zeolite 4X, and from 0.433 to 1.931 mS for the zeolite NC.

### 3.3. Tests on Cement Pastes Modified with Zeolite Admixtures

#### 3.3.1. Calorimetry

**Figure 5:**
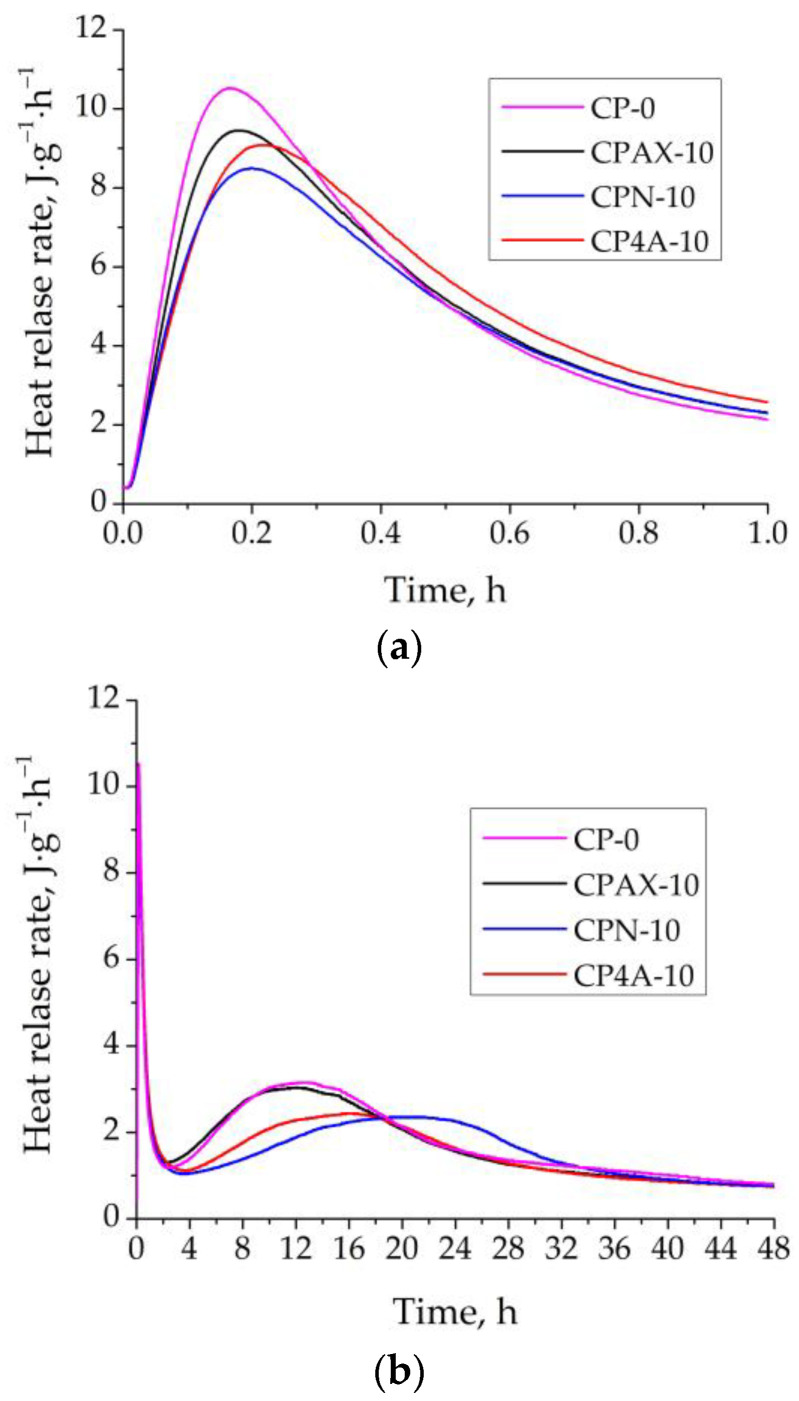
Heat release rate in control cement sample and samples with AX, 4A, and natural zeolites: (**a**) during first hour, (**b**) during 48 h.

Calorimetric investigations were conducted to evaluate the influence of cement replacement by zeolite, to study the hydration process of cement (Figure 5), and to determine the total energy released. The initial increase in heat release corresponds to the wetting process of cement and (C_3_A) hydration. During the cement hydration process, the initial increase in heat release corresponds to the wetting period, which continuously passes to the induction stage, which is characterised by the lack of thermal effects. The sudden increase in heat release is strictly connected to the massive exothermic precipitation of hydrates, followed by further hydration [32]. The results of the replacement of cement by zeolite in the samples show that compared to the control sample, zeolites reduced the wetting heat release rate due to the lower cement amount (dilution effect). The wetting heat release rate for the sample with natural zeolite was reduced by up to 19%compared to the control sample. The replacement of cement by AX zeolite decrease the wetting heat release rate by up to 8%, and the replacement of cement by 4A zeolite, by up to 13%. The same tendencies were observed in [33].

Likewise, it can be seen in Figure 5 that the lower reaction rate delays the dormant and acceleration periods compared to the reference sample, while in the samples, the cement was replaced by 4A zeolite and natural zeolite. The presence of AX zeolite in the sample showed minimal influence on the dormant and acceleration periods in the sample, but showed a slight influence on the control sample. This can be seen by the presence of a mix of different modified zeolites (modified X zeolite; modified A zeolite) with an incompletely crystallised structure and the presence of gibbsite, which can modify C_3_S hydration [22,34]. The main peak (the second peak in Figure 5) can be affected by AFt, C_3_S hydration, the formation of C-S-H, and Ca(OH)_2_. In comparison to the control sample, just using 4A zeolite and natural zeolite significantly decrease heat release rate during the second peak by up to 25%. The maximum heat release rate time for the control sample and sample with AX zeolite was reached after 13 h and 11 h. For the sample with 4A zeolite and natural zeolite, the maximum heat release rate time was reached after 17.8 h and 22.2 h. As was pointed in other studies [22,34,35,36], the presence of natural zeolite leads to lower hydration rates compared to synthetic zeolite.

This indicates that natural zeolite and 4A zeolite prolong the cement hydration process and decrease the heat release rate. The cement hydration process is extended by an additional 5–9 h. It can be stated that newly created AX zeolite, due to the high pH values and the large number of ions in the paste (reflected in the high electrical conductivity), a small acceleration of the cement hydration process and minimally decreases the heat release rate.

**Figure 6:**
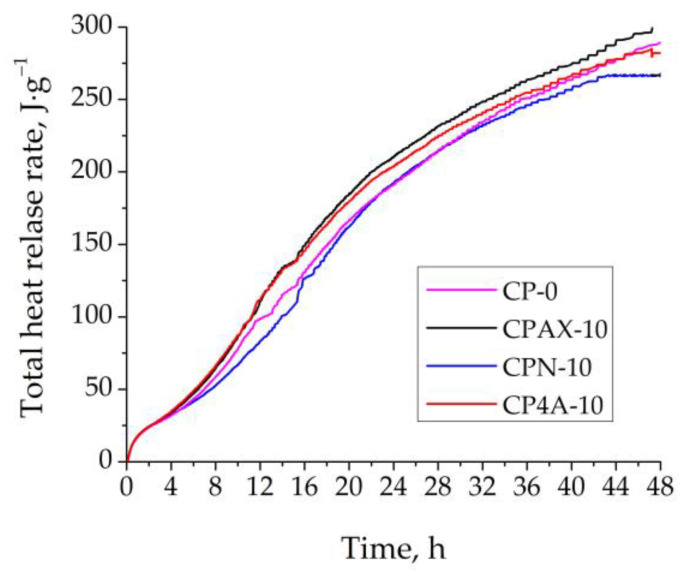
Total heat value during a hydration period of 48h in control cement sample and samples with AX, 4A, and natural zeolites.

The amount of total heat released in the control sample and samples with zeolites can be seen in Figure 6. The replacement of cement by AX zeolite in the samples increases the total heat released by 8.5% after 48 h of hydration compared to control sample (Figure 6). The use of 4A zeolite and increased the total heat value by up to 2%, while the use of natural zeolite decreased it up to 6% compared to the control sample. It can be stated that synthesised AX zeolite released more energy than the pure cement sample, as is proved in [34]. This can be attributed to the higher electrical conductivity and pH of AX zeolite in suspension, as observed in Figure 4. The AX zeolite and 4A zeolite, showing a low pH value in a water solution, can slightly accelerate the cement hydration process. This proves that both AX zeolite and 4A zeolite contribute to the increase in the medium’s pH in the presence of cement minerals [37]. The total heat value after 48 h of hydration supports this hypothesis. It can be concluded that synthesised AX zeolite, due to its higher pH and the number of ions that have passed into the solution from the paste, increases the total heat value in the sample, thereby promoting active cement hydration (more active than in the control sample), and is mostly promising among the other presented zeolites. As we can see from the chemical formula of the zeolites, higher amounts of Na and Al in XA zeolite is presented. In [38,39,40,41] it has been pointed that higher amounts of Na and Al in the material increase it pozzolanic activity.

#### 3.3.2. EXO Profile

**Figure 7:**
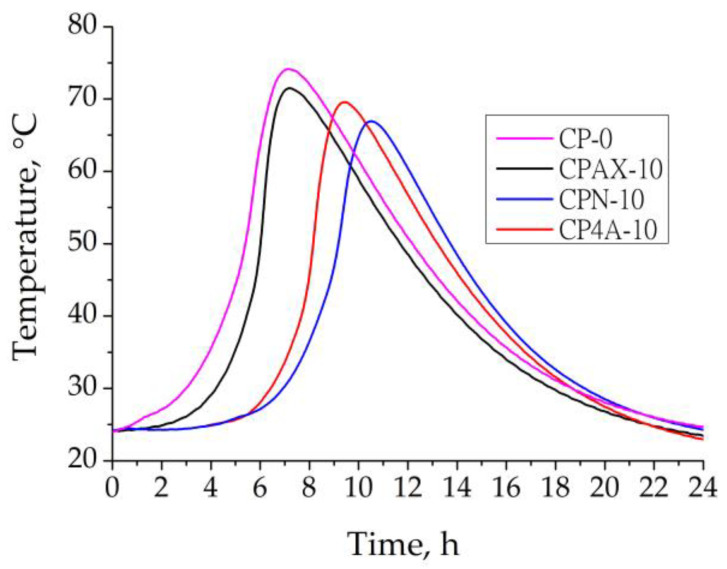
EXO profile of the fresh control cement sample and samples with AX, 4A and natural zeolites.

An EXO profile was created to determine whether the heat emitted during cement hydration affects the temperatures of the pastes with cement replaced by zeolites that were examined (Figure 7).

The test results showed that in the control sample paste, the EXO maximum reaching time was 7.8 h. With the cement replaced by AX zeolite, the EXO maximum reaching time did not change. Apparently, this is due to the highest pH and electrical conductivity values of AX zeolite in suspension. When the 4A zeolite and natural zeolites were used in the paste, the EXO maximum reaching time prolongs from 7.8 to 10.52 h. It is possible that the lower pH and electrical conductivity of 4A zeolite, and especially of natural zeolite suspension, lead to some retardation in the dissolution of cement minerals, which is reflected by an increase in EXO time. The EXO temperature of the control sample paste reached 75.3 °C, but when 10% of the cement was replaced by AX, 4A and natural zeolite, the EXO maximum temperature decreased to 73.2, 70.1 and 67.8 °C, respectively, because of the decrease in cement amount in the sample and the nature of the zeolite used. These results show that natural zeolite mostly decreases the EXO maximum temperature by 10% compared to the effect of pure cement paste on the EXO maximum temperature. The same tendencies were observed in [42], in which the effect of natural and synthesised zeolites on hydration heat was investigated. It seems that AX, with the smallest particle size, does not stop the EXO maximum time, even though it reacts more actively with cement minerals. However, this is not true for 4A and natural zeolite because they have coarser particles, which increases the dilution effect.

#### 3.3.3. Rheological Properties of the Cement Pastes

Concrete mix is special because its properties from the preparation of the mix to the final setting differ. These changes are caused by physical and chemical processes in the mix. The rheological properties of the mix mainly depend on the amount and quality of the cement paste in the mix. Water content also has an important effect as it determines the composition and volume of the liquid phase and the development of traction forces, which determines the binding and workability of the entire system. Cement paste is classified as a structural system, which is characterised by a certain initial strength of the system. The structure of the cement paste is formed due to molecular traction forces acting between particles surrounded by a thin film of water. The film of the water in its liquid phase creates a continuous spatial sheath in the cement paste structure, which gives it plastic-like qualities and creates the conditions for the system to flow under the action of external forces. The initial strength or structural viscosity of the cement paste depends on the concentration of the solid-phase water suspension [43].

Concrete mix viscosity is one of the major parameters that enables the estimation of the preliminary time during which the structure or product will be formed [43,44]. In pumped concrete applications, the delivery of concrete mix to the site may last more than 60 min. The structure or product forming time may last up to 20–30 min depending on technological processes. The hydration processes caused by the cement reacting with water, causing the paste to thicken, and when the critical value of viscosity (12,000 mPas) is reached after a certain time, the mix will no longer be suitable for casting. Therefore, it is important to analyse how each zeolite influences the rheological properties of the cement paste within a period of 0–60 min. Tests with modified cement pastes were conducted in order to find the effect of different types of zeolite on the dynamic viscosity of cement paste. Cement pastes with and without a plasticising admixture added at 0.5% by weight of cement were also tested.

The cement paste viscosity tests showed that the viscosity of the cement paste depends on the amount of cement replaced by zeolite and on type of zeolite used to replace the cement.

**Figure 8:**
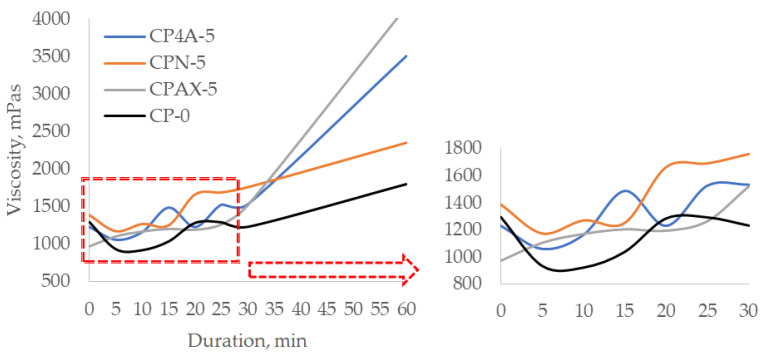
Viscosity change over time of cement paste with a zeolite additive of 5% cement weight.

When zeolite (all three types) is replaced at 5% by weight of cement (Figure 8), during the first 30 min, the viscosity increases from 300 to 550 mPas in the pastes with zeolites, and the viscosity of the control cement paste increases by 40 mPas compared to initial viscosity values. A sharp increase in the viscosity of the cement pastes with zeolite is observed after 30 min. The viscosity of the cement paste increases up to 4160 mPas after 60 min when cement was replaced by zeolite AX. At the same time, in the cement paste in which cement was replaced by 4A zeolite, the viscosity increases up to 3505 mPas, and in case of natural zeolite, the viscosity increases only up to 2351 mPas. It can be noted that the lowest viscosity increase of up to 1800 mPas is seen in the control sample.

**Figure 9:**
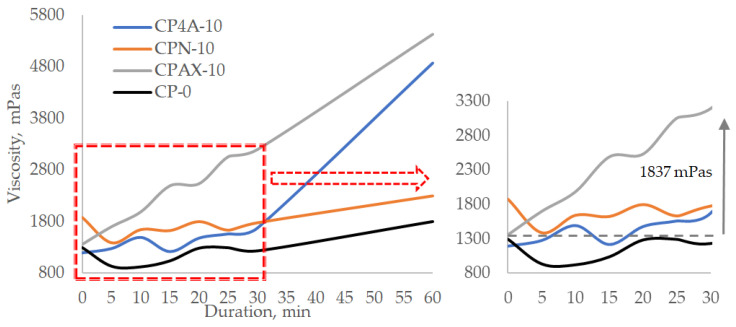
Viscosity change over time of cement paste with a zeolite additive of 10% cement weight.

Tests with cement pastes in which zeolite is replaced at 10% by weight of cement (Figure 9) showed that in the case of zeolite AX the viscosity starts increasing immediately after mixing the paste, and within 15 min, it increases from 1360 to 2493 mPas. The viscosity of the paste continues growing and reaches 3197 mPas after 30 min and 5340 mPas after 60 min. In the case of zeolite 4A, in the first 15 min, the viscosity increases from 1194 to 1217 mPas; after 30 min, it reaches 1561 mPas; and after 60 min, it reaches 4872 mPas. Natural zeolite demonstrated the least effect on viscosity increase after 60 min. The initial viscosity of the paste was relatively high at 1877 mPas. After 15 min, it dropped to 1389 mPas, then an increase was observed after every 5 min. After 30 min, the viscosity went up to 1777 mPas and reached 2298 mPas after 60 min. These results prove that replacing cement with zeolite has a significant effect on the viscosity of the cement paste and that zeolites with a higher pH values accelerate the viscosity increase rate because they hasten the hydration of cement minerals and the precipitation of active hydration products [45,46,47].

**Figure 10:**
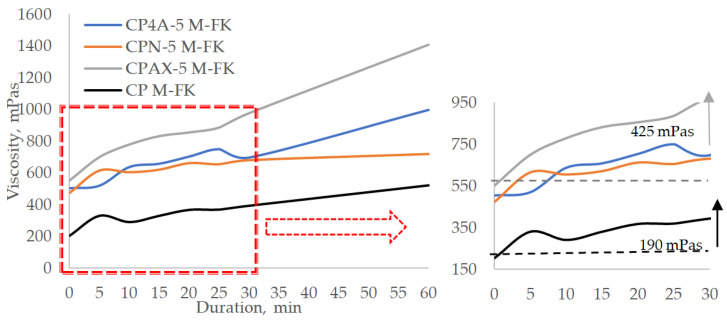
Viscosity change over time of cement paste with a zeolite additive of 5% of cement by weight and 0.5% plasticising admixtures.

**Figure 11:**
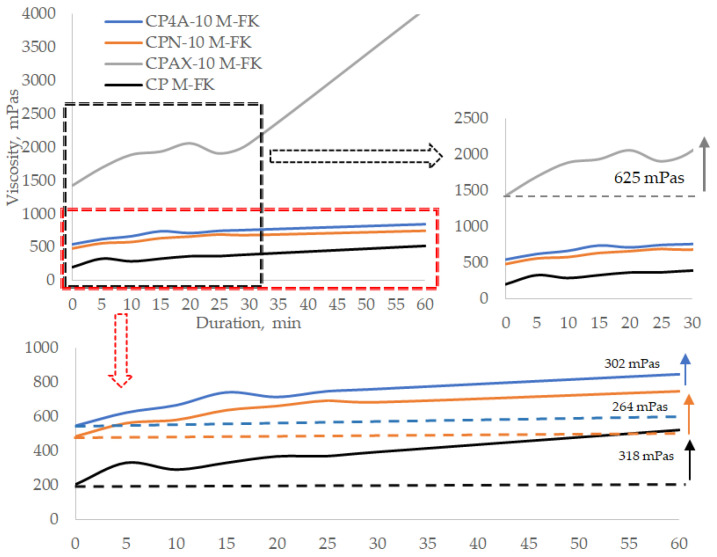
Viscosity change over time of cement paste with a zeolite additive of 10% of cement by weight and 0.5% plasticising admixture.

Tests on cement pastes containing plasticising admixtures (Figure 10 and Figure 11) revealed that the admixture has a significant effect on both the initial viscosity of the paste and the change in viscosity over time. The working mechanism of polycarboxylate resin-based superplasticisers is based on the electrostatic repulsion of cement particles and the additional “ball” effect [48]. This superplasticiser extends the delay of the hydration process. The tests revealed that cement paste viscosity is significantly reduced by adding 0.5% of plasticising admixture.

The analysis of cement paste viscosity values after a certain period of time revealed that cement paste plasticity is lower, compared to plasticising admixture free paste, when 5% of zeolite admixture and plasticising admixture are added; all zeolite admixtures have a similar effect of cement paste viscosity. There is an up-to-5-fold difference in the initial viscosity of the control cement pastes and pastes in which cement was replaced by zeolite, but the earlier observed tendencies remain. The highest viscosity values are observed when cement was replaced by AX zeolite. A 5% replacement of cement by zeolites reduces the viscosity from 1.37 to 2.4 times compared to cement pastes without the plasticising admixture. After 30 min, the viscosity of all cement pastes increases about 200–400 mPas from their initial value. The distribution of modified pastes into higher and lower viscosity groups is observed only after 60 min. After 60 min, the paste modified with AX zeolite reaches a viscosity of 1409 mPas. This viscosity value is 2.9 times lower in pastes without the plasticising admixture. The viscosity of the paste modified with 4A zeolite reaches 998 mPas, and the paste modified with natural zeolite reaches a viscosity of 720 mPas. The obtained results proves that the plasticising admixtures must be added to cement pastes modified with zeolite to improve paste flowability.

A significant increase in viscosity is observed in cement pastes modified with 10% zeolite AX compared to other pastes (Figure 11). The initial viscosity value of this paste is 1431 mPas, and it is higher than the viscosity of the paste without a plasticising admixture. After 30 min, the viscosity of this paste went up to 2056 mPas and reached 4079 mPas after 60 min. Presumably, the selected superplasticiser has no plasticising effect when zeolite AX is used, i.e., the reaction of cement minerals with the plasticising admixture does not produce any film or produces a very thin film that does not prevent water from reaching both zeolite and cement particles and thus causes the viscosity of the paste to increase. This can be explained by the highest Na amount in the zeolite and highest Al/Si ratio, which quickens the creation of newly formed products, with the highest pH value of this zeolite suspension improving cement minerals dissolution [41,49].

The analysis of the change in cement paste viscosity values over time shows that in the first 15 min, the viscosity value in the paste modified with 4A zeolite increases by 50 mPas per 5 min, and in the time span of 15 to 30 min, the viscosity value increases by 15–20 mPas. After 30 min, the viscosity of the paste modified with 4A zeolite is 763 mPas, and in the paste modified with natural zeolite, it reaches 685 mPas. After 60 min, the viscosity of the paste modified with 4A zeolite remains almost the same at 850 mPas, while the viscosity of the paste modified with AX zeolite reaches 4079 mPas, and the viscosity of the paste modified with natural zeolite reaches 749 mPas. The presence of the plasticising admixture in the modified cement paste with 10% zeolites causes a decrease in viscosity by 1.35 times in the AX modified paste, by 5.9 times in the 4A modified paste, and by 2.7 time in the natural zeolite modified paste. Such results suggest that for the paste modified with AX zeolite, the choice of plasticising admixture must be decided very careful.

For control sample paste, this reduction is 4 fold. This viscosity-reducing effect is caused by the reaction of zeolites and the plasticising admixture with cement minerals and the thin film caused by reaction products on the surface of the cement particles [41,49]. This film changes the cement particle surface charge from positive to negative and thus creates repulsion forces between particles. Water penetration into the system is also reduced. This effect, however, is short-term; the surface films are unstable, and water reaches the surface of the cement particle, and thus, the viscosity of the paste increases. At the same time, it can be pointed that AX zeolite provide the highest viscosity of paste due to higher number of ions in the paste, leading to a faster hydration process of cement minerals [22].

#### 3.3.4. Electrical Conductivity and pH Tests in Modified Cement Pastes

The effect of AX zeolite obtained from aluminium fluoride manufacture waste product on cement paste pH and electrical conductivity values was tested in cement pastes modified with different amounts of cement being replaced by zeolite (0%, 5%, 10%). The following pH values of water solutions are known: 12.18 for the cement suspension, 9.93 for the AX zeolite suspension, and 5.05 for the plasticising admixture.

**Figure 12:**
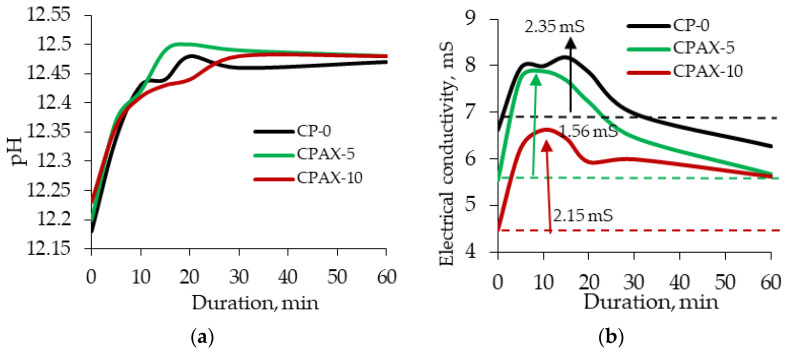
pH and electrical conductivity kinetics of cement paste. (**a**) pH; (**b**) electrical conductivity.

The analysis of the results showed that a slight increase in cement paste pH from 12.18 to 12.25 occurred when the amount of cement replaced by zeolite increased to 10% (Figure 12a). pH measurements after 5 and 10 min showed the same tendencies as with the initial values. After 15 min, a pH value increase in the cement paste modified with 5% zeolite is observed and remains up until 30 min. In the case of the cement paste modified with 10% zeolite, the pH value is the lowest between 10 and 25 min. From the beginning of the test to 30 min, when zeolite ions migrate into the solution (a part of the hydration processes), the pH value of the cement pastes stabilises and remains constant up until 60 min. This results prove that AX zeolite accelerates cement mineral dissolution and increases pH values despite the reduction in cement content. Electrical conductivity tests on cement pastes showed that the initial electrical conductivity values in cement pastes differ (Figure 12b). These differences are caused by the substitution of 10% of cement with AX zeolite in cement pastes. It is known from the results of previous tests that within 24 h, the electrical conductivity in cement changes from 12.26 to 16.43 mS, and from 1.53 to 3.21 mS in the zeolite admixture. The initial electrical conductivity of non-modified cement paste suspension is 6.62 mS. It decreases to 5.53 mS when the paste is modified with 5% zeolite and drops down to 4.47 mS when the paste is modified with 10% zeolite. The electrical conductivity of non-modified cement paste rapidly increases in the first 5 min, remains constant 5 and 20 min, and starts decreasing between 20 and 60 min. In cement paste modified with 5% zeolite, an even higher growth in electrical conductivity is observed during the first 5 min. The downward trend starts at 10 min and continues until the end of the test. In the cement paste modified with 10% zeolite admixture, the electrical conductivity increases up to 6.22 mS after 5 min, with a further increase up to 6.62 mS after 10 min. A rapid drop in electrical conductivity is observed after 15 min, and very similar electrical conductivity values are recorded after 20 and 30 min. The obtained results showed that cement replaced by zeolite at 5% minimally influences the electrical conductivity value, but a 10% replacement can decrease electrical conductivity in the paste by up to 18%. For this reason, it is possible to believe that a greater replacement of cement (more than 10%) by zeolite AX can significantly reduce the electrical conductivity in the paste and significantly inhibit the cement hydration process.

### 3.4. Physical and Mechanical Characteristics of Hardened Modified Cement Paste

**Figure 13:**
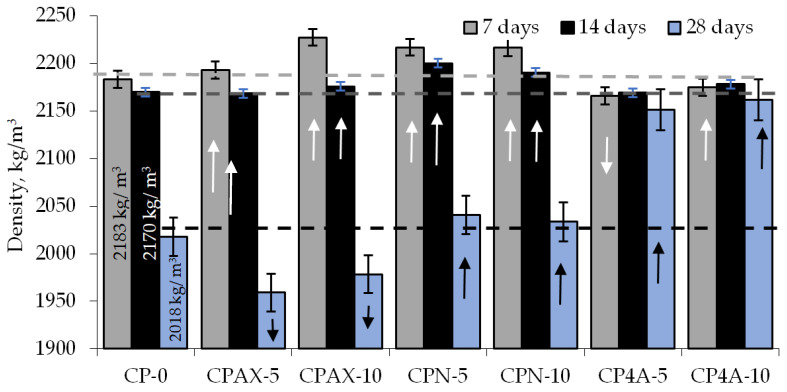
Density of hardened cement paste with zeolite additives.

Seven mix composition mixes were designed: control composition CA-0 and six compositions with three different zeolites. Zeolites were added at 5 and 10% by weight of cement. The results presented in Figure 13 reveal a slight increase in density (1.5%) after 7 days of curing in hardened cement paste modified with 5% natural zeolite. After 14 days of curing, the density increases by 1.4%, and the increase after 28 days was 1.1%. The same trend was observed in hardened cement pastes modified with 10% natural zeolite: 1.5% increase after 7 days of curing, 0.9% increase after 14 days, and 0.8% increase after 28 days [38,41,49].

The density results for the hardened cement paste modified with AX zeolite obtained from aluminium fluoride manufacture waste product are a little higher than those of natural zeolite. The addition of zeolite at 5% caused the density of the samples to increase by 0.4% after 7 days of curing. After 14 days of curing, the density of the modified samples was the same as that of the unmodified samples, and after 28 days of curing, the density reduced by 2.9%. In cement pastes with a zeolite admixture content up to 10%, due to the accelerated creation of cement hydration products, the density increases by 2.0% after 7 days of curing, by 0.2% after 14 days of curing, and reduces by 2.0% after 28 days of curing. The test results showed that natural zeolite and AX zeolite obtained from aluminium fluoride manufacture waste product have little effect on the density of hardened modified cement paste.

**Figure 14:**
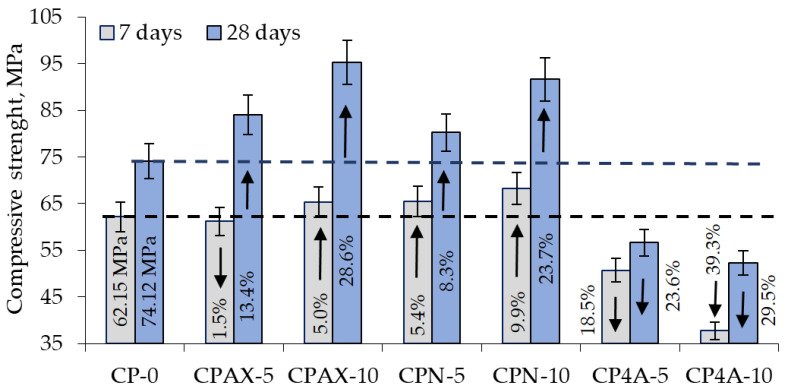
Hardened cement paste with zeolite additive. Compressive strength after 7 and 28 days of hardening.

Figure 14 shows the results of compressive strength tests. Samples modified with zeolite 4A had much lower compressive strength compared to control samples. After 7 days of curing, the compressive strength in samples modified with 5% zeolite admixture was 18.5% lower, and in samples modified with 10% zeolite admixture, the compressive strength was 39.3% lower than in unmodified samples. When the cement was replaced by zeolite 4A, the same is reflected in [50]. The same trend was observed [51] also after 28 days of curing.

After 7 days of curing, the compressive strength of the samples modified with 5% natural zeolite increased by 5.4%, and in the samples modified with 10% natural zeolite, the compressive strength increased by 9.9%. After 28 days of curing, the compressive strength of the samples containing 5% natural zeolite admixture increases by 8.3%, and in the samples containing 10% natural zeolite admixture, the compressive strength increases by 23.7%. Some researchers [38,39,49] argue that synthetic hydrosodalite (zeolite) has a positive effect on the compressive strength of hardened cement pastes, especially at the beginning of cement hydration due to its pozzolanic activity and the formation of aluminate hydrates.

Tests with samples containing AX zeolite obtained from aluminium fluoride manufacture waste product showed that 5% of the admixture caused an insignificant change in compressive strength after 7 days of curing, i.e., a reduction of 1.5%, whereas after 28 days of curing, a 13.4% increase in compressive strength was observed. In samples modified with 10% zeolite, a 5% increase in compressive strength was observed after 7 days of curing, with a further increase in compressive strength by 28.6% after 28 days of curing. The increase in compressive strength can be related to active SiO_2_ and Al_2_O_3_ present in zeolite admixtures [52] as well as to the presence of gibbsite in the system. The presence of aluminate hydrates can provide additional hydration products, such as CSH [22,39].

**Figure 15:**
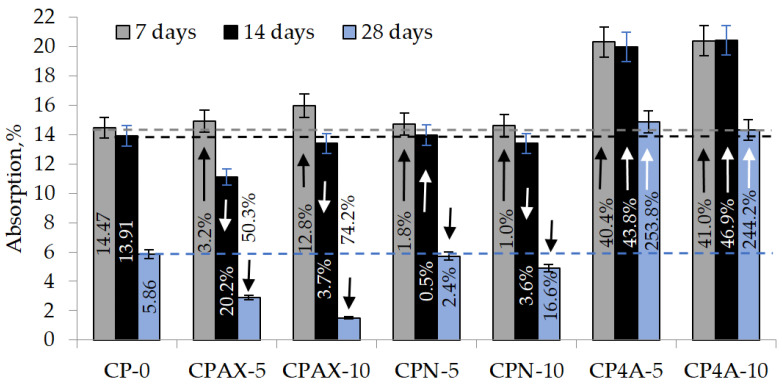
Hardened cement paste with zeolite additives. Absorption value after 7, 14, and 28 days of hardening.

Figure 15 shows the results of water absorption tests. The highest rate of absorption of water was recorded in samples modified with zeolite 4A. After 7 and 14 days of curing, samples modified with 5% and 10% synthetic zeolite 4A had similar rates of water absorption, at above 40%. In samples modified with 5% zeolite, the rate of water absorption changes within the range of 1%. The following changes in the rate of water absorption were observed in samples modified with 10% zeolite: a 1% increase after 7 days of curing, a 3.6% decrease after 14 days of curing, and a 16.6% decrease after 28 days of curing.

After 7 days of curing, the rate of water absorption in samples modified with synthetic zeolite obtained from aluminium fluoride manufacture waste product increased as follows: 3.2% in samples containing 5% zeolite admixture and 10.4% in samples containing 10% zeolite admixture. After 14 days of curing, the rate of water absorption decreased as follows: 20.2% in samples containing 5% zeolite admixture and 3.7% in samples containing 10% zeolite admixture. After 28 days of curing, the rate of water absorption decreased even more. In samples containing 5% zeolite admixture the rate of water absorption was 3.9 times lower compared to the control sample, and in samples containing 10% zeolite admixture, it was more than twice lower compared to the control sample.

## 4. Conclusions

The obtained results lead to the conclusion that newly sintered waste-based zeolite and industrially produced 4A zeolite, due to a higher Al/Na ratio, have higher electrical conductivity and pH values when in suspensions and cement pastes compared to natural zeolite. Synthesised AX zeolite possess 6 times higher electrical conductivity compared to industrially produced 4A zeolite and more than 20 times higher electrical conductivity compared to natural zeolite. The pH value of a Zeolite in a water suspension ranges from alkaline (for AX zeolite and 4Azeolite) to neutral (for natural zeolite). As the consequence, the electrical conductivity values of cement pastes modified with AX, 4A, and natural zeolites are by 31%, 75% and 88.7% lower, respectively, compared to control cement paste. The viscosity, heat evolution, temperature, and hydration process of the cement paste with zeolites mostly depends on its electrical conductivity values in a water suspension.

Due to having the highest Al/Na ratio, pH, and electrical conductivity values, AX zeolite accelerates the maximum heat release rate time by 2 h compared to control sample. In contrast, both 4A zeolite and natural zeolite, characterised by lower pH values and lower electrical conductivity values, prolongs the cement hydration process, and increases the maximum heat release rate time by 27% and 41.4% compared to the control sample. The replacement of cement by 10% AX zeolite in the paste minimally changes heat release rate, whereas 4A zeolite and natural zeolite, due to their lower pH and electrical conductivity, decrease it by 25% and 26.4%, respectively. The Replacement of cement by 10% AX zeolite in the sample increases the total heat by 8.5% after 48 h of hydration. The use of 4A zeolite and natural zeolite decreases the total heat value by up to 2% and 6%, respectively, compared to the control sample. The EXO temperature tests confirm the calorimetry studies and revealed that the replacement of cement by 10% AX zeolite in the cement paste did not influence the EXO maximum reaching time; it is the same as in control sample.

The viscosity of cement paste modified with 10% AX zeolite is up to 2.7 times higher than the same composition with 4A zeolite and natural zeolite. The increase in viscosity of the cement paste modified with AX zeolite is related to its high Al/Na ratio, pH, and electrical conductivity values, which accelerate the precipitation of cement hydration products and increase the viscosity. The increase of viscosity in paste with zeolite 4A is related to the particle size, which reaches 5.38 µm and is more than twice lower than in other zeolites.

Compared to the compositions, in which 10% of the cement was replaced with 4A zeolite or natural zeolite, the replacement of cement by 10% synthetic zeolite AX, due to presence of mineral gibbsite, which speeds up the precipitation of hydration products, such as CSH development, increases the compressive strength after 28 days of curing up to 28.6%, and reduces the water absorption by up to 1.5%.

The tests have proved that newly sintered waste-based zeolite is more effective than industrially produced 4A zeolite and natural zeolite. Newly sintered waste-based zeolite can be used in the development of new, clean, and green construction products. Zeolites increase the durability of these products, i.e., reduce water absorption, and increase compressive strength and density. It can be concluded that synthesised waste-based AX zeolite is cheap because its production is based on waste materials and is mostly promising due to superior properties of created construction materials compared to the other presented zeolites.

## Figures and Tables

**Table 1:** CEM I 42.5 R physical, mechanical, chemical properties and mineral composition.

Properties	CEM I 42.5 R	Chemical Composition, %		Mineral Composition of Clinker, %	
Specific surface, cm^2^/g	4128	SiO_2_	20.7	C_3_S	58.54
Particle density, kg/m^3^	3210	Al_2_O_3_	6.12	C_2_S	15.29
Bulk density, kg/m^3^	1260	Fe_2_O_3_	3.37	C_3_A	10.40
Normal density paste, %	25.4	CaO	63.5	C_4_AF	10.17
Initial setting time, min	142	K_2_O	1.00		
Compressive strength after 7 days, MPa	29.9	SO_3_	0.80		
Compressive strength after 28 days, MPa	55.6	Na_2_O	0.30		
		Other	4.45		

**Table 2:** Chemical composition of zeolites.

Type	SiO_2_	Al_2_O_3_	Fe_2_O_3_	TiO_2_	CaO	MgO	K_2_O + Na_2_O
Clinoptilolite	71.5	13.1	0.9	0.2	2.1	1.07	5.03
AX zeolite	39.3	26.6	-	-	7.5	-	7.20
4A zeolite	34.4	30.3	-	-	-	-	17.2

**Table 3:** Cement paste compositions, 1 m^3^.

Materials	Marks of Mixtures						
	CP-0	CPAX-5	CPAX-10	CPN-5	CPN-10	CP4A-5	CP4A-10
Zeolite, kg	0	58	116	63	126	53	106
Cement, kg	1647	1565	1482	1565	1482	1565	1482
Water, kg	445	445	445	445	445	445	445
Plasticisers, kg	8.20	8.20	8.20	8.20	8.20	8.20	8.20
W/C	0.27	0.28	0.30	0.28	0.30	0.28	0.30

## Data Availability

Not applicable.
